# The Burden of the Perfect Frame: A Scoping Review on Personality and Muscle Dysmorphia

**DOI:** 10.3390/bs16020173

**Published:** 2026-01-26

**Authors:** Valentina Tavoloni, Mariagrazia Di Giuseppe, Marco Innamorati, Marta Mirabella, Vittorio Lingiardi, Laura Muzi

**Affiliations:** 1 Department of History, Humanities, and Society, University of Rome Tor Vergata, 00133 Rome, Italy; tavoloni.valentina@gmail.com (V.T.); mariagrazia.di.giuseppe@uniroma2.it (M.D.G.); innamorati@gmail.com (M.I.); 2 Department of Dynamic and Clinical Psychology, and Health Studies, Faculty of Medicine and Psychology, Sapienza University of Rome, 00185 Rome, Italy; marta.mirabella@uniroma1.it (M.M.); vittorio.lingiardi@uniroma1.it (V.L.); 3 Department of Philosophy, Social Sciences and Education, University of Perugia, Piazza Ermini, 1, 06123 Perugia, Italy

**Keywords:** muscle dysmorphia, muscularity, bigorexia, personality, body image, risk factors

## Abstract

Research on muscle dysmorphia (MD), currently conceptualized as a clinical specifier for body dysmorphic disorder (BDD), is rapidly expanding. Although personality traits and disorders have been proposed as relevant risk factors for the development of BDD, their role in MD remains insufficiently understood. This scoping review aims to synthesize the existing empirical literature on the associations between MD and personality, while identifying key research gaps and clinical challenges. Following the PRISMA-ScR guidelines, a systematic search was conducted across PsycArticles, PubMed, Scopus, Web of Science, and Google Scholar between 1 October and 1 December 2024. A total of 15 studies met the inclusion criteria and were analyzed. Findings highlight the significant contribution of narcissism, neuroticism, and perfectionism to the development and severity of MD. In particular, traits associated with vulnerable narcissism consistently emerged as predictors of MD symptomatology. Sociocultural factors—such as the competitive environment of elite sports and early relational experiences—were also found to interact with personality-based vulnerabilities in shaping the onset and clinical expression of MD. However, most available studies relied on self-report measures, cross-sectional designs, and convenience samples predominantly composed of men, limiting the generalizability of the results. Despite these methodological limitations, this review emphasizes the importance of identifying personality-based vulnerabilities to enhance the understanding of MD and inform the development of person-centered prevention and intervention strategies.

## 1. Introduction

Muscle dysmorphia (MD) is a mental health condition characterized by a persistent preoccupation with the belief that one’s body is not sufficiently lean and muscular, even when the individual may have an objectively muscular physique (Pope et al., 1997). Early studies referred to this type of body image disturbance as “bigorexia” or a “reverse” form of anorexia nervosa (Pope et al., 1993), highlighting its potential connection to feeding and eating disorders (EDs). However, this condition is currently classified as a subtype of body dysmorphic disorder (BDD) in both the *Diagnostic and Statistical Manual of Mental Disorders*, Fifth Edition, Text Revision (DSM-5-TR; American Psychiatric Association, 2022) and the *International Classification of Diseases*, Eleventh Revision (ICD-11; World Health Organization, 2022).

Recent systematic reviews have shown that individuals with MD typically experience a distorted body image, perceiving themselves as small, weak, or insufficiently muscular. Other commonly associated features include body-related anxiety and stress, as well as engagement in problematic behaviors aimed at increasing muscle mass and leanness—such as extreme weightlifting routines, restrictive diets, excessive use of dietary supplements, and the potential use of anabolic–androgenic steroids (Martenstyn et al., 2022; Pope et al., 2017; Tod et al., 2016). These individuals may also avoid situations in which their bodies could be scrutinized by others, and may neglect important social, occupational, or interpersonal responsibilities due to their compulsive need to maintain exercise and diet regimens. Although both men and women can experience MD, existing evidence suggests that males report higher levels of overall MD symptomatology (Lechner et al., 2019).

The etiology and risk factors associated with MD remain insufficiently understood (Tod et al., 2016). Several sociocultural contributors have been proposed, including exposure to media promoting muscular body ideals and the internalization of rigid masculine norms (e.g., Ganson et al., 2023; Giordano et al., 2025), as well as negative social interactions during childhood and adolescence, such as bullying or comparisons with peers and siblings (Edwards et al., 2017). Notably, a recent study identified a higher prevalence of adverse childhood experiences in individuals with MD, including physical and emotional neglect and insecure early attachment (Tingaz, 2020). Other individual-level factors also appear relevant in the development and maintenance of MD, including low self-esteem, emotional dysregulation, and body dissatisfaction (Grieve, 2007). Finally, MD has frequently been associated with pronounced disordered eating symptoms, such as dietary restriction, purging behaviors, and intentional weight loss strategies (Badenes-Ribera et al., 2019; Prnjak et al., 2022).

Despite these promising findings, the role of personality patterns, traits, and disorders in the onset, course, and clinical presentation of MD remains underexplored. This gap in the literature is surprising, considering that personality variables have been established as significant risk factors for BDD from both theoretical and empirical perspectives. For instance, cognitive behavioral models of BDD (e.g., Veale, 2004; Wilhelm, 2006) describe certain personality traits, such as perfectionism, as risk factors. Similarly, while not specifically related to BDD, psychodynamic-oriented approaches outline that clinicians should begin their psychological evaluations by trying to understand the meaning and function of a patient’s symptoms within the broader context of their personality functioning (Westen et al., 2006). Empirical studies have also shown that 57–87% of individuals with BDD meet the criteria for at least one personality disorder, typically among the avoidant, dependent, and obsessive–compulsive types (Cohen et al., 2000; Phillips & McElroy, 2000). Other investigations have reported paranoid, schizotypal (Bellino et al., 2006), and borderline (Neziroglu et al., 1996) personality disorders in patients with BDD. Moreover, research on specific personality traits suggests that individuals with BDD are frequently characterized by high levels of neuroticism and perfectionism—traits closely linked to perceived physical flaws, fear of negative evaluation, anticipatory shame, and pervasive feelings of defectiveness and embarrassment (Pavan et al., 2008; Schieber et al., 2013).

The few studies specifically addressing MD have highlighted a possible association between personality traits and MD symptoms, mirroring findings from the ED literature, which reveals intense self-focus and low self-esteem as prominent features (Lewis-Smith et al., 2016; Mirabella et al., 2023; Muzi et al., 2021). Notably, theory-driven accounts developed specifically for MD point to the relevance of personality-linked individual differences (e.g., low self-esteem, negative affectivity, perfectionism) as factors that may increase vulnerability to MD by shaping body dissatisfaction, distorted self-evaluation, and the internalization of a muscular ideal (Grieve, 2007). Furthermore, pathological narcissism—particularly the vulnerable subtype—has been linked to behaviors commonly seen in MD, such as compulsive exercise, steroid use, and engagement in risky activities aimed at compensating for deep-seated inadequacy and shame (Bruno et al., 2014; Giancola et al., 2024; Hill, 2016). However, the findings remain mixed. For example, Kuennen and Waldron (2007) found no significant correlations between pathological narcissism and MD symptoms, whereas other studies (e.g., Murray & Baghurst, 2013; Rodrigue et al., 2018) have identified associations between MD symptoms and perfectionism, with the latter appearing to underlie rigid attitudes and behaviors in pursuit of an ideal body shape.

The existing literature on the relationship between personality and MD remains methodologically heterogeneous. Studies vary substantially in the personality constructs examined, the psychometric instruments employed, and the operationalization of MD symptoms. This fragmentation in the literature limits the ability to draw cumulative conclusions about which personality features are most consistently associated with MD. It also limits understanding of how personality traits and/or disorders may relate to the onset, maintenance, or clinical course of MD, including potential implications for clinically relevant outcomes such as treatment response, motivation for change, and resistance to therapeutic interventions. A consolidated synthesis to map and compare findings across studies is currently lacking. Such a synthesis may help to clarify consistent patterns of association and inform future research priorities. In turn, a more precise understanding of the links between personality and MD may support the development of targeted prevention strategies and individualized psychotherapeutic approaches.

### Review Questions

The present scoping review aims to systematically map the literature on the role of personality in MD to inform clinical practice and identify knowledge gaps that may guide future research. Specifically, the review seeks to address the following questions:

(a) Which personality traits and/or personality disorders are most commonly associated with MD?
(b) Are there psychosocial or cultural factors that moderate or mediate the relationship between personality and MD?
(c) What clinical implications and research challenges have been identified by the authors of relevant studies?
(d) Which aspects of the relationship between personality and MD remain underexplored and warrant further investigation?

## 2. Materials and Methods

The scoping review was conducted in accordance with the Preferred Reporting Items for Systematic Reviews and Meta-Analyses extension for Scoping Reviews (PRISMA-ScR) guidelines, which informed the search strategy, methodological framework, and reporting procedures (Tricco et al., 2018). The search strategy was developed collaboratively by the authors through preliminary meetings held in September 2024. No restrictions were applied to the publication date, in order to capture the full breadth of empirical findings on the relationship between personality and MD.

### 2.1. Study Selection Criteria

Articles were included in the scoping review if they met the following criteria:

(a) Published in English in peer-reviewed journals;
(b) Involving participants exhibiting either subclinical or clinical symptoms of MD (also referred to as reverse anorexia or bigorexia), assessed using validated measures; and
(c) Providing a formal assessment of personality traits, styles, or disorders using validated measures.

The following types of publications were excluded: non-English language studies, qualitative studies, book chapters, review articles, author responses, correction notices, and conference proceedings. No restrictions were applied to the publication date.

### 2.2. Search Strategy

A comprehensive electronic search was conducted across the PsycArticles, PubMed, Scopus, and Web of Science databases. In addition, Google Scholar was consulted to identify potentially relevant additional records. The literature search was conducted in the “title” and “abstract” fields (or equivalent fields, depending on the database), using the following Boolean strategy:

*(“body dysmorphia” OR “body dysmorphic disorder” OR “muscle dysmorphic” OR “muscle dysmorphia” OR “bigorexia” OR “reverse anorexia” OR “Muscle Appearance Satisfaction Scale” OR “Muscle Dysmorphic Disorder Inventory” OR “Muscle Dysmorphia Inventory” OR “Muscle Dysmorphia Questionnaire” OR “muscularity”) AND (“personality” OR “personality disorder” OR “narcissistic” OR “temperament”).*

The complete, database-specific search strings are provided in Supplementary Materials S2.

All identified records were uploaded to Rayyan, an online platform designed to support systematic reviews, for duplicate identification/removal and screening management. Duplicate entries were identified automatically by Rayyan and then manually verified by a reviewer to ensure accuracy.

**Figure 1:**
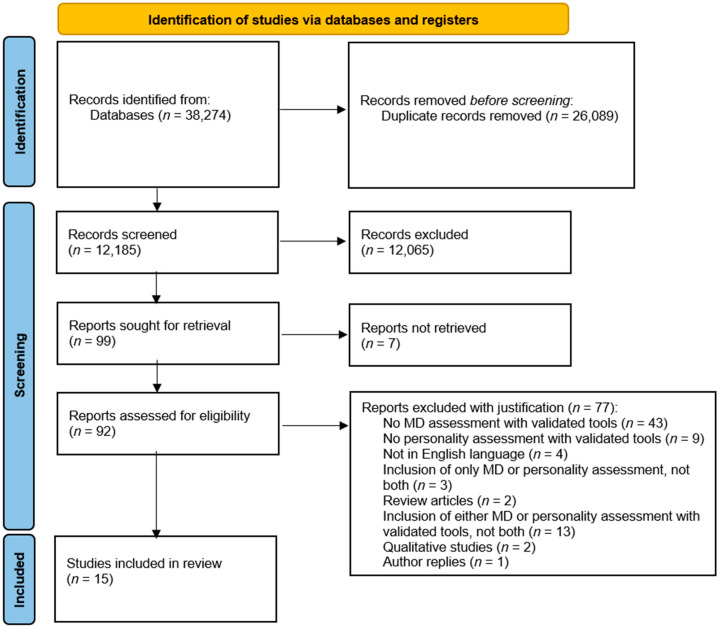
Flow chart of the selection and screening process in the scoping review.

The screening process was conducted independently by two authors, with support from a graduate research assistant who assisted with article selection and data extraction. Discrepancies regarding study eligibility were resolved through discussion and consultation with a third author. Additionally, inter-rater agreement was calculated for each screening phase. The screening and selection process was conducted in two stages: (i) title/abstract screening, followed by (ii) full-text assessment of potentially eligible reports. The software reported an inter-rater agreement of 81% for the title/abstract screening phase and 78% for the full-text screening phase. The PRISMA flowchart is presented in Figure 1.

### 2.3. Study Selection

The initial search yielded a total of 38,274 records. Among the initial records, 26,089 were identified as duplicates and therefore excluded. This high number of duplicates reflects substantial overlap across the databases searched and repeated indexing of the same records across sources. The remaining 12,185 records were screened based on titles and abstracts. At this stage, 12,086 records were excluded for reasons including the absence of a validated assessment of MD and/or personality, non-English language, non-empirical designs (e.g., commentary or opinion pieces), or other predefined exclusion criteria.

A total of 99 reports were sought for retrieval. Seven full texts could not be retrieved, and 92 full-text articles were assessed for eligibility. In the final phase, full-text reports were assessed to confirm eligibility and relevance to the scope of the review; 92 reports were assessed in a full-text format, and 15 met all predefined inclusion criteria (Figure 1). The remaining 77 full-text reports were excluded after retrieval for the following mutually exclusive reasons: publication type (author reply, *n* = 1; qualitative study, *n* = 2; review article, *n* = 2); absence of a validated assessment of MD (*n* = 43); absence of a validated assessment of personality (*n* = 9); validated measurement for only one construct (MD or personality; *n* = 13); assessment of only one construct (MD or personality), but not both (*n* = 3); and non-English language (*n* = 4). Table 1 summarizes the main characteristics of the included studies.

## 3. Results

### 3.1. Characteristics of the Included Studies

All of the included studies employed a cross-sectional design and were conducted with moderate to large sample sizes, ranging from 100 to 566 participants (*M* = 290.7), drawn from non-clinical populations. Eleven studies (Bégin et al., 2019; Benford & Swami, 2014; Boulter et al., 2023; Collis et al., 2016; Davis et al., 2005; Dèttore et al., 2020; Harmancı & Okray, 2021; Hater et al., 2021; Kandemir et al., 2024; Littrell et al., 2021; Merhy et al., 2023) included exclusively male samples, three studies (Boulter & Sandgren, 2022; Maurin et al., 2024; Zarei, 2020) reported mixed-gender samples, and one study (Hater et al., 2021) involved only a female sample.

The average age of participants across the studies was 25.18 years. The youngest sample was reported by Zarei (2020), which included only adolescents (*M_age_* = 16.31), while the oldest sample was observed in Szymczak et al. (2023), with a mean age of 36.76 years. The studies were conducted across several countries. Three were conducted in Canada (Bégin et al., 2019; Benford & Swami, 2014; Davis et al., 2005); one in Italy (Dèttore et al., 2020); two in Turkey (Harmancı & Okray, 2021; Kandemir et al., 2024); two in the United Kingdom (Benford & Swami, 2014; Boulter & Sandgren, 2022); two in the United States (Littrell et al., 2021; Szymczak et al., 2023); and one each in Lebanon (Merhy et al., 2023), Australia (Collis et al., 2016), and Iran (Zarei, 2020). In addition, one was a multinational study conducted jointly in the United Kingdom and the United States (Boulter et al., 2023), and another spanned Spain and Germany (Hater et al., 2021).

Participants’ ethnicities varied across the studies and included Caucasian and African American (Littrell et al., 2021); Canadian (Benford & Swami, 2014); Turkish (Hater et al., 2021); French-Canadian (Maurin et al., 2024); Spanish and German (Hater et al., 2021); and British, Asian, and African-Caribbean (Benford & Swami, 2014). Eight of the included studies (Boulter & Sandgren, 2022; Boulter et al., 2023; Collis et al., 2016; Davis et al., 2005; Dèttore et al., 2020; Harmancı & Okray, 2021; Merhy et al., 2023; Zarei, 2020) did not report participants’ ethnic backgrounds.

A range of instruments was used to assess both MD and personality dimensions. Six studies (Boulter & Sandgren, 2022; Boulter et al., 2023; Davis et al., 2005; Harmancı & Okray, 2021; Kandemir et al., 2024; Merhy et al., 2023) employed the Muscle Dysmorphic Disorder Inventory (MDDI; Hildebrandt et al., 2004), while five studies (Benford & Swami, 2014; Hater et al., 2021; Maurin et al., 2024; Szymczak et al., 2023; Zarei, 2020) used the Drive for Muscularity Scale (DMS; McCreary & Sasse, 2000). Notably, Bégin et al. (2019) administered both measures. Two additional instruments were also used to assess MD: the Muscle Dysmorphia Inventory (MDI; Short, 2006), employed by Collis et al. (2016), and the Muscle Dysmorphia Questionnaire (MDQ; Grieve et al., 2014), used by Littrell et al. (2021).

For personality assessment, the most frequently used instrument was the Narcissistic Personality Inventory (NPI; Raskin & Hall, 1979; Raskin & Terry, 1988), with variations including the 16-item version (Boulter & Sandgren, 2022) and the full NPI-40 (Collis et al., 2016; Davis et al., 2005; Dèttore et al., 2020; Harmancı & Okray, 2021; Hater et al., 2021; Littrell et al., 2021; Szymczak et al., 2023). Additionally, the Hypersensitive Narcissism Scale (HSNS; Hendin & Cheek, 1997) was employed to assess vulnerable narcissism in four studies (Hater et al., 2021; Littrell et al., 2021; Szymczak et al., 2023; Zarei, 2020). An alternative measure of vulnerable narcissism was the Five-Factor Narcissism Inventory—Short Form (FFNI-SF; Sherman et al., 2015), which was administered by Boulter et al. (2023) and Hater et al. (2021). To assess broader personality traits, Benford and Swami (2014) used the Neuroticism-Extraversion-Openness Five-Factor Inventory (NEO-FFI; Costa & McCrae, 1992), while Davis et al. (2005) applied the Eysenck Personality Questionnaire–Revised (EPQ-R; Eysenck & Eysenck, 1993). Regarding the assessment of perfectionism, two studies (Davis et al., 2005; Kandemir et al., 2024) employed the Multidimensional Perfectionism Scale (MPS; Hewitt et al., 1991). The Multidimensional Inventory of Perfectionism in Sport (MIPS; Stoeber et al., 2006) was used by Maurin et al. (2024), the Big Three Perfectionism Scale (BTPS; Smith et al., 2016) by Merhy et al. (2023), and the Almost Perfect Scale–Revised (APS-R; Slaney et al., 2001) by Zarei (2020).

Key findings regarding the associations between MD and personality traits are summarized in the following sections of this scoping review.

### 3.2. Which Personality Traits and/or Personality Disorders Are Most Commonly Associated with Muscle Dysmorphia?

Eight studies (Bégin et al., 2019; Benford & Swami, 2014; Boulter et al., 2023; Dèttore et al., 2020; Harmancı & Okray, 2021; Hater et al., 2021; Littrell et al., 2021; Szymczak et al., 2023) reported significant associations between narcissistic traits and MD symptoms. Bégin et al. (2019) found that narcissistic vulnerability moderated the relationship between drive for muscularity, negative affect, and MD symptoms. Specifically, men exhibiting high levels of both narcissistic vulnerability and drive for muscularity reported greater negative affect and an increased risk of developing MD symptoms. Similarly, Boulter and Sandgren (2022) found that vulnerable narcissism was significantly and positively associated with MD, whereas grandiose narcissism showed no significant relationship.

Extending these findings, Boulter et al. (2023) emphasized the central role of vulnerable narcissism in men as a mediator of the association between paternal relationship quality and MD symptoms. Specifically, paternal relationships characterized by emotional distancing and criticism were found to contribute to internalized feelings of unworthiness and heightened preoccupation with muscular bodily appearance. Additionally, Dèttore et al. (2020) reported higher levels of narcissism among competitive bodybuilders compared to non-competitive counterparts, suggesting an interaction between narcissistic traits and the expression of MD symptomatology.

Interestingly, Harmancı and Okray (2021) reported that Functional Impairment (FI)—a subscale of the Muscle Dysmorphic Disorder Inventory (MDDI) that reflects the extent to which individuals maintain their exercise routines despite negative consequences—was positively correlated with narcissism. This finding suggests that individuals with higher narcissistic traits may be more likely to persist in compulsive workout behaviors, even when such behaviors interfere with daily functioning or psychological well-being.

Hater et al. (2021) further refined the narcissism–MD association in a sample of women by considering the trifaceted model of narcissism: agentic, antagonistic, and neurotic. Neurotic narcissism—marked by hypersensitivity, shame, and fragile self-esteem—emerged as a significant predictor of both drive for muscularity and drive for thinness. Notably, while the association with drive for thinness was partially mediated by the importance attributed to physical appearance, no such mediation effect was observed for the drive for muscularity.

Additionally, both Littrell et al. (2021) and Szymczak et al. (2023) examined facets of grandiose narcissism in relation to muscularity-related variables. Littrell et al. (2021) identified a significant association between drive for muscularity and specific facets of grandiose narcissism, particularly those related to self-enhancement and admiration seeking. Moreover, the facets of authority and entitlement were positively associated with the persistence dimension of MD symptoms. However, no significant relationship was found between grandiose narcissism and overall MD symptomatology.

Szymczak et al. (2023) examined the relationship between specific facets of narcissism (i.e., agentic extraversion, antagonism, narcissistic neuroticism) and body image concerns—including drive for muscularity, current and desired muscularity, drive for thinness, and ED symptoms—in both men and women. Agentic extraversion was negatively associated with drive for muscularity, potentially serving as a protective factor by promoting a more balanced self-image. In contrast, neurotic narcissism was positively associated with MD symptoms. Regarding sex differences, men were more strongly characterized by antagonism and reported both a greater drive for muscularity and a higher ideal muscularity compared to women. Conversely, women showed a stronger drive for thinness than men.

Finally, among the studies that investigated the role of narcissism, Collis et al. (2016) was the only one that reported no significant association between narcissistic traits and MD symptoms. However, the study found that negative body attitudes—particularly weight salience and self-criticism—were the strongest predictors of MD.

Two studies (Benford & Swami, 2014; Davis et al., 2005) identified neuroticism as a significant predictor of drive for muscularity. Benford and Swami (2014) found that neuroticism was positively associated with drive for muscularity and negatively associated with body appreciation, highlighting the dual influence of this personality trait on body image and MD symptomatology. Similarly, Davis et al. (2005), who examined personality correlates of drive for muscularity in men, also found that neuroticism significantly predicted this MD variable. These findings suggest that the emotional hypersensitivity characteristic of neuroticism may contribute to a more critical self-evaluation of one’s muscular body.

Perfectionistic traits have also been examined in relation to MD (Hater et al., 2021; Maurin et al., 2024; Merhy et al., 2023; Zarei, 2020). Davis et al. (2005) found that individuals with elevated anxiety, pronounced perfectionism, and heightened concern with physical appearance and fitness were more likely to report increased levels of drive for muscularity. Similarly, Maurin et al. (2024) demonstrated that, within sport-specific contexts, perfectionism significantly predicted MD-related symptoms. Notably, gender differences were observed: among male athletes, perfectionism was more directly associated with muscularity-focused concerns, whereas in female athletes, it was more strongly linked to general body dissatisfaction.

Merhy et al. (2023), in examining the association between perfectionism and MD among athletes, identified orthorexia nervosa as a mediator of this relationship. Specifically, they proposed the existence of a psychological triad in which perfectionism—particularly in its rigid and self-critical forms—intensifies MD symptoms through the idealization of “perfect nutrition” that is central to orthorexia.

Lastly, Kandemir et al. (2024) identified perfectionism as a key mediating variable in the association between vulnerable narcissism and the risk of developing MD. Their findings suggest that individuals with elevated levels of vulnerable narcissism may be more prone to engage in perfectionistic behaviors, which, in turn, may heighten the pursuit of an idealized muscular physique.

### 3.3. Are There Psychosocial or Cultural Factors That Moderate or Mediate the Relationship Between Personality and Muscle Dysmorphia?

Several sociocultural factors have been identified as interacting with personality-based vulnerabilities in contributing to the onset and expression of MD symptoms. Drawing on Stice’s dual pathway model, Bégin et al. (2019) proposed an etiological framework in which sociocultural pressures—particularly the internalization of a lean and muscular male body ideal, alongside culturally sanctioned norms of masculinity—foster increased body dissatisfaction. This dissatisfaction, in turn, may give rise to maladaptive behaviors characteristic of MD, including rigid dietary control, compulsive exercise, and the use of muscle-enhancing substances. Importantly, these sociocultural pressures appear to interact with specific personality traits, such as vulnerable narcissism, thereby amplifying vulnerability to MD symptomatology.

Two studies (Benford & Swami, 2014; Hater et al., 2021) have also demonstrated associations between the internalization of idealized body standards and personality traits such as perfectionism and neuroticism, both of which have been linked to increased drive for muscularity and greater body dissatisfaction. Additionally, Boulter et al. (2023) highlighted the influence of early relational experiences, particularly adverse relationships with paternal figures during childhood and adolescence. Such experiences were associated with lower self-esteem and the adoption of compensatory behaviors centered on muscularity in adulthood. These findings suggest that early deficits in emotional validation—especially from paternal figures—may contribute to the development of vulnerable narcissism, particularly among males, thereby heightening the need for external validation through physical appearance and muscular enhancement.

The competitive environment of elite sports has also been identified as a salient sociocultural context in which MD symptomatology may intersect with personality traits (Harmancı & Okray, 2021; Hater et al., 2021; Maurin et al., 2024). As noted by Maurin et al. (2024), individuals engaged in high-level athletic competition are frequently subjected to intense performance- and appearance-related pressures, which can contribute to elevated body dissatisfaction. Their findings suggest that the structural demands and social climate of elite sports function as both direct and indirect stressors, particularly when athletes conflate self-worth and social acceptance with physical performance and appearance. Notably, elite athletes reported significantly higher levels of both body dissatisfaction and perfectionistic traits compared to their non-elite counterparts. Within this framework, perfectionism—especially when coupled with the internalization of external appearance standards—appears to amplify maladaptive striving toward an idealized muscular physique, reinforcing rigid training regimens and heightened preoccupation with muscularity.

### 3.4. What Clinical Implications and Research Challenges Have Been Identified by the Authors of Relevant Studies?

The findings from the reviewed studies underscore several key clinical implications for a person(ality)-centered approach to the prevention, assessment, and treatment of MD and related symptoms. A consistent theme across the literature is the importance of systematically assessing personality traits—particularly perfectionism, neuroticism, and narcissism—given their significant role in the onset and maintenance of MD symptomatology (Benford & Swami, 2014; Boulter et al., 2023; Collis et al., 2016; Davis et al., 2005; Dèttore et al., 2020; Harmancı & Okray, 2021; Kandemir et al., 2024; Maurin et al., 2024; Zarei, 2020).

Moreover, in light of the multidimensional nature of narcissism and its nuanced associations with MD, several studies (Boulter & Sandgren, 2022; Littrell et al., 2021; Szymczak et al., 2023) emphasize the need to move beyond a unitary conceptualization and differentiate among its various facets—namely, grandiose and vulnerable narcissism, as well as agentic, antagonistic, and neurotic subtypes (Boulter & Sandgren, 2022; Hater et al., 2021; Littrell et al., 2021; Szymczak et al., 2023). In particular, vulnerable and neurotic narcissism—characterized by emotional instability, hypersensitivity to criticism, and fragile self-esteem—appear to represent central contributors to MD symptoms (Boulter & Sandgren, 2022; Hater et al., 2021; Littrell et al., 2021), elevating the drive for thinness and muscularity (Hater et al., 2021; Szymczak et al., 2023). These traits may increase individuals’ reliance on external validation through physical appearance, thereby intensifying body dissatisfaction and engagement in risk-related behaviors.

Prevention strategies and therapeutic interventions should therefore prioritize the enhancement of emotional regulation capacities, the reduction of shame, and the development of stable, internalized sources of self-worth (Boulter & Sandgren, 2022; Szymczak et al., 2023). At the same time, traits commonly associated with grandiose narcissism—such as agentic extraversion and antagonism—may also contribute to symptom expression, particularly by fueling excessive striving for admiration through adherence to idealized body standards. These dimensions should not be overlooked when designing individualized treatment plans (Littrell et al., 2021; Szymczak et al., 2023).

In this vein, Dèttore et al. (2020), in their study comparing competitive and non-competitive bodybuilders, identified two distinct personality-based profiles: one characterized by narcissistic traits and pride, and the other by depressive traits and shame. These findings underscore the importance of individualized treatment approaches tailored to the patient’s underlying personality configuration. Specifically, interventions for narcissistic profiles should target perfectionistic standards and dependence on external validation, whereas treatment for more vulnerable, depressive presentations should prioritize shame reduction and the enhancement of self-esteem regulation.

Moreover, the study by Kandemir et al. (2024) highlighted the conceptual and clinical overlap between MD and EDs, noting that both conditions share core vulnerability factors such as vulnerable narcissism and perfectionistic traits. From a clinical perspective, these similarities support the integration of MD within established ED assessment and treatment frameworks—particularly those targeting maladaptive perfectionism, distorted body image, and emotional dysregulation.

Several of the reviewed studies (e.g., Collis et al., 2016; Harmancı & Okray, 2021; Maurin et al., 2024; Zarei, 2020) also advocated for the implementation of targeted screening and psychological support within high-risk settings such as bodybuilding gyms, weight training facilities, and competitive athletic environments. These contexts—especially for adolescents and young adults—may intensify sociocultural pressures and reinforce appearance-focused beliefs, particularly in individuals already predisposed by personality vulnerabilities.

Finally, the gender differences reported by Maurin et al. (2024)—specifically, a significantly higher drive for muscularity among men and elevated levels of socially prescribed perfectionism and negative body evaluations among women—underscore the need for gender-sensitive intervention models. These findings suggest that gender may moderate the psychological mechanisms linking personality traits to MD symptomatology and should thus be considered in both prevention and treatment planning.

### 3.5. Which Aspects of the Relationship Between Personality and Muscle Dysmorphia Remain Underexplored and Warrant Further Investigation?

Beyond the primary findings outlined in the reviewed studies, substantial gaps persist in our understanding of the relationship between personality and MD symptomatology, highlighting several critical areas for future investigation.

First, existing research has primarily focused on subclinical personality traits—particularly narcissistic features—while largely neglecting the potential contribution of full-threshold personality disorders to the etiology, presentation, and maintenance of MD. For example, despite considerable attention to narcissistic traits in relation to MD (Bégin et al., 2019; Boulter & Sandgren, 2022; Boulter et al., 2023; Harmancı & Okray, 2021; Hater et al., 2021; Littrell et al., 2021; Szymczak et al., 2023), the role of narcissistic personality disorder (NPD) as a diagnostic entity remains underexplored. Future studies should explicitly examine whether individuals with NPD are at heightened risk for developing MD, and whether the co-occurrence of these conditions is associated with more severe or treatment-resistant presentations.

Additionally, while recent studies have emphasized the role of vulnerable narcissism in MD, its emotional underpinnings—particularly shame and pride—warrant further examination. These self-conscious emotions may represent important mediators in the link between narcissistic vulnerability and maladaptive muscular-focused behaviors.

Another notably underinvestigated area concerns the role of emotional dysregulation. Although studies exploring neuroticism (Benford & Swami, 2014; Harmancı & Okray, 2021) have highlighted the contribution of negative affectivity to MD symptomatology, the broader construct of emotion regulation difficulties—central to borderline personality disorder (BPD)—has yet to be systematically examined in this context. Exploring the extent to which features associated with BPD (e.g., affective instability, impulsivity, identity disturbance) are implicated in MD could offer a more nuanced understanding of its developmental trajectories and inform more tailored clinical interventions.

Notably, future research should move beyond the examination of isolated personality traits and adopt a more comprehensive approach to personality organization and functioning. This broader perspective may help to elucidate how personality structure shapes bodily self-perception and subjective experiences, thereby contributing to the onset and maintenance of MD. In line with this framework, a key emerging area of investigation involves the intersection between EDs, MD, and personality traits or disorders (Hater et al., 2021; Littrell et al., 2021; Maurin et al., 2024; Merhy et al., 2023). Given the overlapping symptomatic expressions (e.g., body dissatisfaction, compulsive behaviors, distorted body image), further research is warranted to clarify the shared and distinct personality-based vulnerabilities underlying these conditions.

Importantly, none of the reviewed studies specifically targeted clinical populations, representing a significant limitation. Future research should prioritize clinical samples to better understand the presentation of MD in real-world clinical contexts and to identify challenges related to its assessment, diagnosis, and treatment. Moreover, developmental aspects remain markedly underexplored. There is a pressing need for longitudinal studies including younger cohorts to examine how early-emerging personality traits interact with developmental influences (e.g., media exposure, peer relationships, emerging self-concept) to increase the risk for MD later in life (Zarei, 2020).

Finally, it is worth noting that no standardized or empirically validated treatment protocol for MD currently exists, and research on therapeutic interventions remains scarce. Establishing and evaluating targeted interventions that address both personality-based vulnerabilities and sociocultural pressures is a critical next step for the field.

Informed by these insights, a critical challenge for future research lies in clarifying the role of personality variables in the treatment and clinical management of MD. Understanding how specific personality traits and disorders influence treatment responsiveness, the therapeutic alliance, and long-term outcomes could significantly enhance intervention strategies. Moreover, the reviewed literature is predominantly based on cross-sectional designs, limiting causal inferences and clinical generalizability. To address these limitations, future studies should prioritize longitudinal research designs, recruit more diverse and representative samples—encompassing a broader range of ages, cultural backgrounds, and gender identities—and incorporate more comprehensive and multidimensional personality assessments. Such advancements are essential to better capture the complexity of MD and inform the development of personalized, effective treatment approaches.

## 4. Discussion

The present scoping review aimed at synthesizing the empirical literature investigating the role of personality in the etiology, symptomatology, and treatment implications of muscle dysmorphia (MD). The findings underscore the substantial influence of narcissism, neuroticism, and perfectionism in the development and severity of this condition, while indicating consistent associations, as well as direct and indirect effects between personality traits and MD-related behaviors. Overall, the findings suggest that personality characteristics may provide a framework within which muscularity concerns and related behaviors are organized and experienced. From this perspective, MD symptoms appear to be meaningfully embedded in broader personality-based patterns, rather than representing isolated behavioral manifestations. Nevertheless, the findings warrant cautious interpretation, as the predominantly cross-sectional design of the reviewed studies precludes robust inferences regarding causal relationships.

Several studies (Bégin et al., 2019; Benford & Swami, 2014; Boulter et al., 2023) highlighted a consistent association between vulnerable narcissism and MD symptomatology, in line with the broader literature linking narcissistic traits to body image disturbances (Carrotte & Anderson, 2019; Purton et al., 2018; Swami et al., 2015). Collectively, this body of work suggests that individuals with vulnerable narcissism—often described as “thin-skinned,” following Rosenfeld’s conceptualization (Rosenfeld, 1987)—may be particularly prone to body dysmorphic symptoms due to their low self-esteem, heightened sensitivity to evaluation, and chronic need for external validation. Within this framework, the pursuit of a hyper-muscular physique may serve as a defensive strategy: an attempt to “thicken the skin,” regulate fragile self-worth, and buffer against perceived inadequacies.

Conversely, grandiose narcissism has demonstrated weak or even negative associations with MD symptomatology. Among the reviewed studies, only Littrell et al. (2021) and Szymczak et al. (2023) explicitly examined the role of specific facets of grandiose narcissism. Littrell et al. (2021) reported that traits such as authority and entitlement were positively associated with the persistence dimension of MD symptoms, yet not with overall MD severity. Similarly, Szymczak et al. (2023) found that agentic extraversion—a facet characterized by assertiveness, confidence, and leadership—was negatively associated with the drive for muscularity, potentially suggesting a protective effect. Antagonism, in contrast, was more prevalent among men and associated with elevated muscularity ideals.

These findings align with prior research (Gordon & Dombeck, 2010; Morrison et al., 2004; Swami et al., 2015) suggesting that grandiose narcissism may exert a generally protective influence against body dissatisfaction. For instance, Carrotte and Anderson (2018) found that grandiose traits predicted lower levels of body shame and weight-related concerns. One possible explanation is that individuals with grandiose narcissism may derive their self-worth primarily from non-physical domains (e.g., status, success, social dominance), thereby reducing the motivational drive to alter or enhance their physical appearance.

However, the positive association between specific grandiose traits (e.g., authority, entitlement, antagonism) and the drive for muscularity may reflect the symbolic value that a muscular physique holds in signaling dominance, power, and social status. In this context, muscularity does not serve to compensate for inner insecurity—as observed in vulnerable narcissism—but rather functions as a means to reinforce and display a superior self-image.

Perfectionistic traits also emerged as significant contributors to higher MD symptomatology, often expressed through compulsive exercise, rigid dietary control, and persistent muscularity-focused preoccupations (Kandemir et al., 2024; Maurin et al., 2024; Merhy et al., 2023; Zarei, 2020). Notably, Merhy et al. (2023) identified orthorexic tendencies as a partial mediator in the relationship between perfectionism and MD, suggesting that the pursuit of a “pure” or “disciplined” body—initially framed as health-oriented—may evolve into pathological muscularity concerns. These findings underscore the multidimensional nature of perfectionism, which includes both adaptive components (e.g., high personal standards) and maladaptive aspects (e.g., self-critical evaluative concerns). The latter, in particular, appear to reinforce rigid ideals of bodily control, thereby exacerbating vulnerability to MD symptoms.

Neuroticism also emerged as a significant risk factor for MD. Studies by Benford and Swami (2014) and Davis et al. (2005) demonstrated that elevated levels of neuroticism are associated with an increased drive for muscularity and decreased body appreciation. These findings are consistent with the broader literature (Dionne & Davis, 2004; Martin & Racine, 2017) identifying neuroticism and emotional instability as key vulnerabilities for maladaptive body-related cognitions and behaviors. Notably, individuals high in neuroticism may engage in compulsive exercise or rigid dietary control as strategies for emotion regulation (Grenon et al., 2016; Murray et al., 2012), suggesting that such behaviors may serve both to mitigate negative affect and reinforce body image concerns.

The reviewed studies also suggest that the association between personality traits and MD symptomatology may differ substantially by gender. In male populations, narcissistic traits (e.g., entitlement, exhibitionism, antagonism, agentic extraversion, neurotic narcissism) were more strongly associated with drive for thinness, while antagonism showed a particularly robust correlation with the drive for muscularity (Szymczak et al., 2023). These traits may reflect internalized masculine ideals centered on dominance and power, with muscularity serving as a way to assert superiority, rather than compensate for insecurity.

In contrast, among female samples, traits such as leadership and authority were more closely linked to disordered eating symptoms, whereas antagonism was paradoxically associated with lower levels of current body fat (Szymczak et al., 2023). Such differences may reflect gendered body ideals: while culturally reinforced norms emphasize thinness and self-restraint among women (often limiting the social legitimacy of overt expressions of dominance), masculine norms more frequently valorize size, strength, and muscularity. Importantly, vulnerable narcissism emerged as a robust transdiagnostic predictor of MD symptomatology—particularly among women (Hater et al., 2021)—especially when physical appearance constituted a core aspect of self-worth.

Similarly, in a large mixed-gender sample of athletes, Maurin et al. (2024) found that perfectionism predicted muscularity-related concerns in men (particularly those engaged in team, resistance, or weight-class sports), whereas in women it was more closely associated with general body dissatisfaction and thinness-oriented striving. These findings support the hypothesis that sociocultural and sport-specific norms shape the expression of perfectionistic striving in gendered ways. Such gendered pathways raise important questions about whether existing conceptualizations of MD—developed predominantly in male samples—adequately reflect the experiences of women presenting with muscularity-oriented concerns. This underscores the need for assessment tools and intervention strategies that are sensitive to gender differences, not only in terms of shared vulnerabilities (e.g., perfectionism, fragile self-esteem), but also in relation to the distinct symbolic meanings that muscularity may hold across genders.

The findings also suggest that specific personality traits do not merely serve as static risk factors, but they may also interact dynamically with social and contextual pressures to shape the manifestation of MD symptoms. In this vein, several studies (e.g., Bégin et al., 2019; Benford & Swami, 2014; Boulter et al., 2023; Harmancı & Okray, 2021) have shown that sociocultural ideals of muscularity and contextual influences (e.g., media exposure, competitive environments) interact with individual traits such as perfectionism, neuroticism, and vulnerable narcissism, thereby amplifying vulnerability to MD. This interaction seems particularly pronounced in appearance-focused contexts, such as elite sports or fitness subcultures, where external pressures intensify the internal drive for bodily control and enhancement (Dèttore et al., 2020; Harmancı & Okray, 2021; Maurin et al., 2024).

Among the various sociocultural factors, the quality of early familial relationships appears particularly significant. Boulter et al. (2023) emphasized that paternal rejection and lack of emotional attunement during childhood may contribute to low self-esteem, fostering compensatory behaviors centered on physical appearance—especially in men, for whom the drive for muscularity may function as a symbolic strategy to restore a sense of adequacy. These findings are consistent with those of Grenon et al. (2016), who highlighted the importance of attachment in shaping body image. Specifically, recollections of maternal figures as less caring were directly associated with poorer body image, whereas paternal lack of care was indirectly linked to body dissatisfaction through increased attachment anxiety and greater internalization of media ideals.

Despite these insights, there are several limitations to this scoping review. First, the literature search was conducted up to December 2024, thereby excluding more recent studies. Given the rapid development of research on MD, studies conducted after this date may include emerging evidence and further refine the current understanding of MD. In addition, because narcissistic traits represent a frequently examined personality dimension in relation to MD, this construct was included as a specific search term. While this choice helped to ensure adequate coverage of this area of research, it may also have resulted in a greater retrieval of studies focused on narcissism compared to other traits. Future reviews may therefore benefit from adopting a broader set of trait-specific personality keywords to expand coverage across additional personality dimensions.

Notably, none of the reviewed studies included participants with severe or functionally impairing MD symptoms, thereby limiting the generalizability of findings to clinical populations. Furthermore, the majority of the included studies focused on male samples, with only three employing mixed-gender samples (Boulter & Sandgren, 2022; Maurin et al., 2024; Szymczak et al., 2023) and just one study involving only female participants (Hater et al., 2021). Given growing evidence that the internalization of muscular body ideals and elevated body dissatisfaction are increasingly prevalent among women (Jürgensen et al., 2025), future research should explore how MD-related symptomatology and associated personality traits manifest in female populations, who may exhibit distinct psychological vulnerabilities and clinical features.

Of particular concern, despite accumulating evidence linking traits such as perfectionism, narcissism, and neuroticism to the development and maintenance of MD, none of the studies included evaluated the efficacy of interventions specifically targeting these personality traits in individuals with MD symptomatology. This gap highlights the need for future research incorporating personality-informed interventions, which may enhance treatment responsiveness and improve long-term outcomes.

## 5. Conclusions

The present scoping review highlights the substantial role of personality variables—particularly vulnerable narcissism, neuroticism, and perfectionism—in the onset and clinical expression of muscle dysmorphia (MD). The findings call for a shift in the conceptualization of MD, moving beyond a narrowly body image-focused framework toward understanding it as a condition embedded within the broader structure of the person(ality).

In this light, the drive for muscularity, preoccupation with physical inadequacy, body image concerns, and associated behaviors may not be mere cognitive or behavioral symptoms but rather expressions of enduring dispositional traits, including affective instability, shame proneness, fragile self-esteem, negative affectivity, and rigid perfectionistic defenses (Di Giuseppe & Lingiardi, 2023; Tavoloni et al., 2024). These vulnerabilities, when interacting with sociocultural pressures—including the internalization of cultural ideals related to muscularity and performance—may intensify body dissatisfaction and reinforce MD symptomatology.

Clinically, this perspective underscores the importance of early identification of personality configurations that confer heightened risk and supports the development of interventions that go beyond symptom reduction. Specifically, treatment efforts should aim to strengthen self-esteem regulation, foster identity integration, and address dysfunctional beliefs associated with narcissistic vulnerability and maladaptive perfectionism.

Future research should prioritize longitudinal, clinically grounded investigations to explore how personality traits interact with sociocultural influences and developmental trajectories in shaping MD onset and maintenance. Embedding personality within comprehensive etiological models may facilitate a transition from symptom-oriented to person-centered conceptualizations of MD, ultimately enhancing assessment accuracy and informing more nuanced, individualized, and transformative psychosocial interventions.

## Figures and Tables

**Table 1:** Characteristics of included studies and key findings.

Authors, Year	Country	Study Design	Sample Size	Participants	Muscle Dysmorphia Measures	Personality Variables and Measures	Key Findings	Limitations
Bégin et al. (2019)	Canada	Cross-sectional	*N* = 386	Mean age = 22.24, *SD* = 4.39 Gender = assigned male Non-clinical population (college students, employees)	Drive for Muscularity Scale (DMS); Muscle Dysmorphic Disorder Inventory (MDDI)	Narcissism, PNI	Narcissistic vulnerability was positively associated with MD symptoms (MDDI-Total: *r* = 0.46, *p* < 0.001; subscales *r* = 0.25–0.35, all *p* < 0.001) and moderated the negative affect pathway linking drive for muscularity to MD symptoms (significant conditional indirect effect only at higher narcissistic vulnerability: IE = 0.037, 95% CI [0.006, 0.082]).	Exclusive reliance on self-report measures; specific sources of social influence were not taken into account; sample consisted almost exclusively of White men recruited from a university setting.
Benford and Swami (2014)	UK	Cross-sectional	*N* = 509	Mean age = 25.18, *SD* = 8.28 Gender = assigned male Non-clinical population	Drive for Muscularity Scale (DMS)	Neuroticism, NEO-FFI	In a sample of men, neuroticism was moderately associated with drive for muscularity (*r* = 0.29, *p* < 0.001) and uniquely predicted drive for muscularity beyond body mass index and subjective social status (*β* = 0.29, *p* < 0.001).	Exclusive reliance on self-report measures; wide age range of participants (i.e., 18–59 years); use of the NEO-FFI limited the analysis to domain-level scores; indirect effects of other relevant variables were not taken into account.
Boulter and Sandgren (2022)	UK	Cross-sectional	*N* = 336	Mean age = 26.35, *SD* = 8.20 Gender = 52% assigned female, 48% assigned male Non-clinical population (college students, employees)	Muscle Dysmorphic Disorder Inventory (MDDI)	Grandiose narcissism, NPI-16; vulnerable narcissism, HSNS	Vulnerable narcissism showed a positive association with overall MD symptoms (*r* = 0.19, *p* < 0.01) and remained a significant predictor even when controlling for demographics and training-related covariates (*β* = 0.19, *p* < 0.01), whereas grandiose narcissism showed a small positive bivariate association with MD symptoms (*r* = 0.14, *p* < 0.05) but was not a significant predictor in the adjusted model.	Risk of shared method variance due to the exclusive reliance on cross-sectional self-report measures; indirect effects of other relevant variables not taken into account.
Boulter et al. (2023)	UK, US	Cross-sectional	*N* = 503	Mean age = 28.5, *SD* = 9.6 Gender = assigned male Non-clinical population	Muscle Dysmorphic Disorder Inventory (MDDI)	Narcissism, FFNI-SF	MD symptoms were positively associated with vulnerable narcissism (*r* = 0.47, *p* < 0.01), whereas the association with grandiose narcissism was non-significant; vulnerable narcissism also mediated the association between a poorer relationship with one’s father and MD symptoms (negative indirect effect via vulnerable narcissism: *β* = −0.11, 95% CI [−0.16, −0.05]), consistent with path estimates showing a negative association between relationship-with-father scores and vulnerable narcissism (*β* = −0.26, 95% CI [−0.30, −0.11]) and a positive association between vulnerable narcissism and MD symptoms (*β* = 0.51, 95% CI [0.36, 0.67]).	Exclusive reliance on self-report measures; assessment of the paternal relationship was conducted retrospectively, focusing exclusively on the father, thereby excluding mothers and/or substitutive figures for the paternal role.
Collis et al. (2016)	Australia	Cross-sectional	*N* = 117	Mean age = 26.02, *SD* = 8.16 Gender = assigned male Non-clinical population (currently training, no longer weight training, former weight trainers, never engaged in weight training)	Muscle Dysmorphia Inventory (MDI)	Narcissism, NPI-40	Narcissistic traits were not significantly associated with MD symptomatology (Spearman’s *ρ* = 0.14, *p* = 0.146); additionally, narcissism did not differ between current and former weight trainers (*t*(104) = 1.68, *p* = 0.096), and MD scores did not differ by training status (*t*(104) = 0.131, *p* = 0.896).	Exclusive reliance on self-report measures; only two of the nine components of Grieve’s (2007) model of MD considered; participant groups based on self-reported frequency of weight training; small sample size in some of the frequency categories.
Davis et al. (2005)	Canada	Cross-sectional	*N* = 100	Mean age = 22.8, *SD* = 3.3 Gender = assigned male Non-clinical population (college students)	Drive for Muscularity Scale (DMS)	Narcissism, NPI-40; Neuroticism scale of EPQ-R; Perfectionism, scale of MPS	Drive for muscularity was positively associated with neuroticism (*r* = 0.27, *p* < 0.01) and self-oriented perfectionism (*r* = 0.31, *p* < 0.01); neuroticism (*b* = 0.25, *p* = 0.003) and self-oriented perfectionism (*b* = 0.19, *p* = 0.022) remained significant predictors (adjusted *R*^2^ = 0.40), whereas narcissism was not significantly associated with drive for muscularity.	Exclusive reliance on self-report measures; moderate sample size composed exclusively of men recruited in a university setting.
Dèttore et al. (2020)	Italy	Cross-sectional	*N* = 178	Mean age = 30, *SD* = 4 Gender = assigned male Non-clinical population (competitive and non-competitive body builders, non-training men)	Muscle Dysmorphic Disorder Inventory (MDDI)	Narcissism, NPI-40	Competitive bodybuilders showed higher narcissistic traits than other groups (*F* = 37.27, *p* < 0.001, partial η^2^ = 0.30) and higher MD symptoms than controls (*F* = 38.24, *p* < 0.001, partial η^2^ = 0.30); narcissism was positively associated with MD symptoms only among competitive bodybuilders (narcissism × group interaction: *F* = 4.09, *p* = 0.018; within competitive bodybuilders: *β* = 0.53, *p* = 0.006).	Exclusive reliance on self-report measures; restricted number of participants within each group.
Harmancı and Okray (2021)	Turkey	Cross-sectional	*N* = 128	Mean age = 26.60, 22.71, *SD* = 4.40, 2.08 Gender = assigned male Non-clinical population (bodybuilders, sedentary individuals)	Muscle Dysmorphic Disorder Inventory (MDDI)	Narcissism, NPI-40	In male bodybuilders versus sedentary men, bodybuilders reported higher MD symptoms overall (*Z* = −5.081, *p* < 0.001) and higher narcissistic traits (*Z* = −2.435, *p* = 0.015); however, within bodybuilders, narcissistic traits did not significantly predict MD symptom dimensions.	Exclusive reliance on self-report measures; sample included only bodybuilders and sedentary individuals.
Hater et al. (2021)	Spain, Germany	Cross-sectional	*N* = 566	Mean age = 32.26, 28.18, *SD* = 10.18, 10.92 Gender = assigned female Non-clinical population	Drive for Muscularity Scale (DMS)	Grandiose narcissism, NPI-40, NARQ; vulnerable narcissism, HSNS, FFNI-SF	Within the trifaceted model of narcissism, encompassing agentic, antagonistic, and neurotic dimensions, drive for muscularity was positively associated with neurotic narcissism (*r* = 0.26, *p* < 0.01) and antagonistic narcissism (*r* = 0.28, *p* < 0.01), whereas its association with agentic narcissism was non-significant; only neurotic narcissism uniquely predicted drive for muscularity (*β* = 0.42, *p* = 0.025), while antagonistic and agentic narcissism showed no incremental effects.	Exclusive reliance on self-report measures; effect of narcissism on drive for muscularity was examined in only one of the two analyzed samples.
Kandemir et al. (2024)	Turkey	Cross-sectional	*N* = 135	Mean age = 24.99 *SD* = 5.38 Gender = assigned male Non-clinical population (gym members)	Muscle Dysmorphic Disorder Inventory (MDDI)	Narcissism, PNI-40; perfectionism, MPS	Vulnerable narcissism predicted MD risk status (*B* = −0.016, *p* = 0.011), but this direct effect became non-significant after accounting for perfectionism (*B* = −0.010, *p* = 0.110), with a significant indirect effect via perfectionism (indirect effect = −0.005, 95% CI [−0.013, −0.002]; mediation path *B* = −0.020, *p* = 0.004).	Exclusive reliance on self-report measures; sample included only gym-going men, who may be considered a high-risk population for MD.
Littrell et al. (2021)	US	Cross-sectional	*N* = 173	Mean age = 20.67, *SD* = 6.4 Gender = assigned male Non-clinical population (college students)	Muscle Dysmorphia Questionnaire (MDQ); Drive for Muscularity Scale (DMS)	Grandiose narcissism, NPI-40; vulnerable narcissism, HSNS	Overall MD symptomatology was not associated with grandiose narcissism, but was positively associated with vulnerable (hypersensitive) narcissism (*r* = 0.20, *p* = 0.015). At the grandiose-narcissism facet level, the most consistent associations involved Authority and Entitlement: Authority was negatively related to Muscularity Drive (*r* = −0.22, *p* < 0.01) and Body Anxiety (*r* = −0.17, *p* < 0.05), but positively related to Increased Muscularity (*r* = 0.15, *p* < 0.01), Compulsion (*r* = 0.21, *p* < 0.01), and Persistence (*r* = 0.22, *p* < 0.01); Entitlement was negatively related to Muscularity Drive (*r* = −0.21, *p* < 0.01) and positively related to Preoccupation (*r* = 0.21, *p* < 0.01), Compulsion (*r* = 0.22, *p* < 0.01), Social Sacrifice (*r* = 0.23, *p* < 0.01), Body Anxiety (*r* = 0.16, *p* < 0.05), and Persistence (*r* = 0.27, *p* < 0.01).	Exclusive reliance on self-report measures; included male students recruited from undergraduate psychology courses; 62 participants (35.8%) did not report their age.
Maurin et al. (2024)	Canada	Cross-sectional	*N* = 254	Mean age = 18.08, *SD* = 2.34 Gender = assigned female 64.5%, assigned male 35.5% Non-clinical population (athletes)	Drive for Muscularity Scale (DMS)	Perfectionism, MIPS	Male sex at birth (vs. female) was a significant predictor of higher drive for muscularity in the adjusted regression model (*β* = −0.24, *p* ≤ 0.001); additionally, perfectionistic aspirations during training were significant positive predictors of both drive for thinness (*β* = 0.30, *p* ≤ 0.001) and drive for muscularity (*β* = 0.23, *p* ≤ 0.001) in the final models.	Exclusive reliance on self-report measures; highly specific sample characteristic (i.e., Francophone Canadian NextGen athletes); low response rate (i.e., 33%).
Merhy et al. (2023)	Lebanon	Cross-sectional	*N* = 396	Mean age = 25.39, *SD* = 4.96 Gender = assigned male Non-clinical population (college students)	Muscle Dysmorphic Disorder Inventory (MDDI)	Perfectionism, BTPS	MD symptom severity showed positive correlations with rigid (*r* = 0.31), self-critical (*r* = 0.37), and narcissistic (*r* = 0.25) perfectionism. Indirect effects of perfectionism on MD symptom severity via orthorexia nervosa were supported for rigid (0.22, 95% BCa CI [0.13, 0.33]), self-critical (0.14, [0.07, 0.21]), and narcissistic perfectionism (0.24, [0.15, 0.33]); via eating attitudes, indirect effects were supported for rigid (−0.18, [−0.30, −0.07]) and self-critical (−0.10, [−0.17, −0.03]) but not narcissistic perfectionism (−0.05, [−0.11, 0.001]).	Exclusive reliance on self-report measures; sample consisted exclusively of men recruited from a university setting; response rate not reported; inclusion of non-validated scales in the Lebanese context (e.g., MDDI).
Szymczak et al. (2023)	US	Cross-sectional	*N* = 430	Mean age = 36.76, *SD* = 10.08 Gender = assigned male 64%, assigned female 36% Non-clinical population	Drive for Muscularity Scale (DMS)	Agentic extraversion and antagonism, NARQ; narcissistic neuroticism, HSNS; agentic extraversion and antagonism, NPI-40	Drive for muscularity showed positive associations with multiple narcissism dimensions, including antagonism (*r* = 0.73, *p* < 0.01), agentic extraversion (*r* = 0.59, *p* < 0.01), narcissistic neuroticism (*r* = 0.69, *p* < 0.01), exhibitionism/entitlement (*r* = 0.49, *p* < 0.01), and leadership/authority (*r* = 0.22, *p* < 0.01); drive for muscularity correlations were comparable across men and women (e.g., antagonism: 0.77 vs. 0.68, *z* = 1.89; agentic extraversion: 0.62 vs. 0.54, *z* = 1.04; narcissistic neuroticism: 0.68 vs. 0.70, *z* = −0.38; all n.s.).	Exclusive reliance on self-report measures; broad age range of participants (18–72 years); data collected during the COVID-19 pandemic; risk of false positives or the amplification of significant findings due to numerous pairwise comparisons.
Zarei (2020)	Iran	Cross-sectional	*N* = 150	Mean age = 16.31, *SD* = 5.37 Gender = assigned male Non-clinical population (high school students)	Drive for Muscularity Scale (DMS)	perfectionism, APS-R	Drive for muscularity was negatively correlated with self-esteem (*r* = −0.38, *p* < 0.01) and positively correlated with maladaptive perfectionism (*r* = 0.32, *p* < 0.01) and interpersonal sensitivity (*r* = 0.25, *p* < 0.01); in multiple regression, self-esteem (*β* = −0.26, *p* < 0.001), maladaptive perfectionism (*β* = 0.18, *p* = 0.02), and interpersonal sensitivity (*β* = 0.16, *p* = 0.04) were significant predictors of drive for muscularity, explaining 52% of the variance (adjusted *R*^2^ = 0.52).	Exclusive reliance on self-report measures; included only male adolescents with at least 1 year of bodybuilding experience.

**Note.** MD = muscle dysmorphia; NEO-FFI = Neuroticism-Extraversion-Openness-Five-Factor Inventory (Costa & McCrae, 1992); PNI = Pathological Narcissism Inventory (Pincus et al., 2009); NPI-16 = Narcissistic Personality Inventory (Ames et al., 2006); HSNS = Hypersensitive Narcissism Scale (Hendin & Cheek, 1997); FFNI-SF = Five Factor Narcissism Inventory Short-Form (Sherman et al., 2015); NPI-40 = Narcissistic Personality Inventory (Raskin & Terry, 1988; Raskin & Hall, 1979); EPQ-R = Eysenck Personality Questionnaire-Revised (Eysenck & Eysenck, 1993); MPS = Multidimensional Perfectionism Scale (Hewitt et al., 1991); NARQ = Narcissistic Admiration and Rivalry Questionnaire (Back et al., 2013); MIPS = Multidimensional Inventory of Perfectionism in Sport (Stoeber et al., 2006); BTPS = Big Three Perfectionism Scale (Smith et al., 2016); APS-R = Almost Perfect Scale-Revised Form (Slaney et al., 2001).

## Data Availability

The data that support the findings of this study are available upon reasonable request from the corresponding author.

## Supplementary Materials

The following supporting information can be downloaded at: https://www.mdpi.com/article/10.3390/bs16020173/s1, File S1: Preferred Reporting Items for Systematic reviews and Meta-Analyses extension for Scoping Reviews (PRISMA-ScR) Checklist. File S2: Search strategy.

## Abbreviations

The following abbreviations are used in this manuscript:

MD	Muscle dysmorphia
BDD	Body dysmorphic disorder
ON	Orthorexia nervosa
EDs	Eating disorders
PRISMA-ScR	Preferred Reporting Items for Systematic Reviews and Meta-Analyses Scoping Review
MDDI	Muscle Dysmorphic Disorder Inventory
MDD	Muscle dysmorphic disorder
NPD	Narcissistic personality disorder
BPD	Borderline personality disorder
